# Unusual Pulmonary Manifestation in a Child With Congenital Mitral Stenosis: A Case Report

**DOI:** 10.7759/cureus.80575

**Published:** 2025-03-14

**Authors:** Ola Alhalabi, Ahmed Abushahin, Atqah Abdulwahab

**Affiliations:** 1 Pediatric Pulmonology, Sidra Medicine, Doha, QAT

**Keywords:** congenital mitral valve stenosis, fatiguability, pediatric surgery, pulmonary hypertension, respiratory difficulty, secondary pulmonary hemosiderosis

## Abstract

Congenital mitral valve stenosis (MVS) in children is usually isolated. However, it is, in rare cases, associated with secondary pulmonary hemosiderosis. It is hypothesized that secondary pulmonary hemosiderosis results from chronic pulmonary venous congestion and repeated microvascular hemorrhage due to elevated left atrial pressure. We describe the case of a six-year-old child with congenital severe MVS who developed coexisting pulmonary hypertension and hemosiderosis. The child was successfully managed with surgical repair of MVS through papillary muscle splitting and commissurotomy. This case illustrates the rare association between MVS and pulmonary hemosiderosis in a child with easy fatiguability and heart failure symptoms.

## Introduction

Mitral valve stenosis (MVS) is an uncommon heart defect. Rheumatic MVS is the most common cause worldwide, while congenital MVS is rare. MVS leads to post-capillary hypertension due to elevated left atrial pressure and pulmonary venous congestion. Over time, this condition results in vascular changes, endothelial dysfunction, and reflex vasoconstriction, culminating in a combined pre- and post-capillary pulmonary hypertension phenotype [1]. Pulmonary parenchymal manifestations of mitral stenosis include pulmonary edema, diffuse alveolar hemorrhage, and secondary pulmonary hemosiderosis [2].

Secondary pulmonary hemosiderosis related to congenital MVS is an uncommon lung disorder in children that presents as diffuse parenchymal infiltrates on chest radiography, hemoptysis, and anemia [3-5]. According to previous reports, up to 16% of persons with MVS will develop the illness [6]. Furthermore, modest deconditioning and a progressive reduction in exercise tolerance may result from pulmonary venous hypertension as well as hemodynamic consequences of MVS and secondary pulmonary hypertension [7] Treatment options for MVS include valve repair or valve replacement [8].

The present case highlights the significance of early detection and surgical intervention, which can prevent irreversible pulmonary vascular remodeling and subsequent right heart dysfunction. 

## Case presentation

A six-year-old boy presented to the emergency room (ED) with lethargy, acutely developing bilateral periorbital edema, and gradual onset of respiratory difficulties. He reported no fever, sore throat, cough, chest pain, hemoptysis, joint pain or swelling, skin rash, or urinary symptoms. When first assessed, he was found to be awake, afebrile, and experiencing mild to moderate respiratory distress. He was also tachypneic, breathing at a rate of 30 breaths per minute, and hypoxic (arterial oxygen saturation (SaO2) 90%) when breathing room air. The patient had a heart rate of 130 beats per minute, sinus tachycardia, normotensive. Physical examination was notable for right-sided heart failure as hepatosplenomegaly of 2 cm below the right costal margin with abdominal distension and pedal edema. He had normal first and second heart sounds. Grade 4 pan-systolic murmur was heard at the left lower sternal border, gallop rhythm. There was no physical finding of collagen diseases, vasculitis, or nephrotic syndrome. His past medical history was remarkable for tonsillitis at the age of four years, and he reported a negative anti-streptolysin test. 

**Figure 1:**
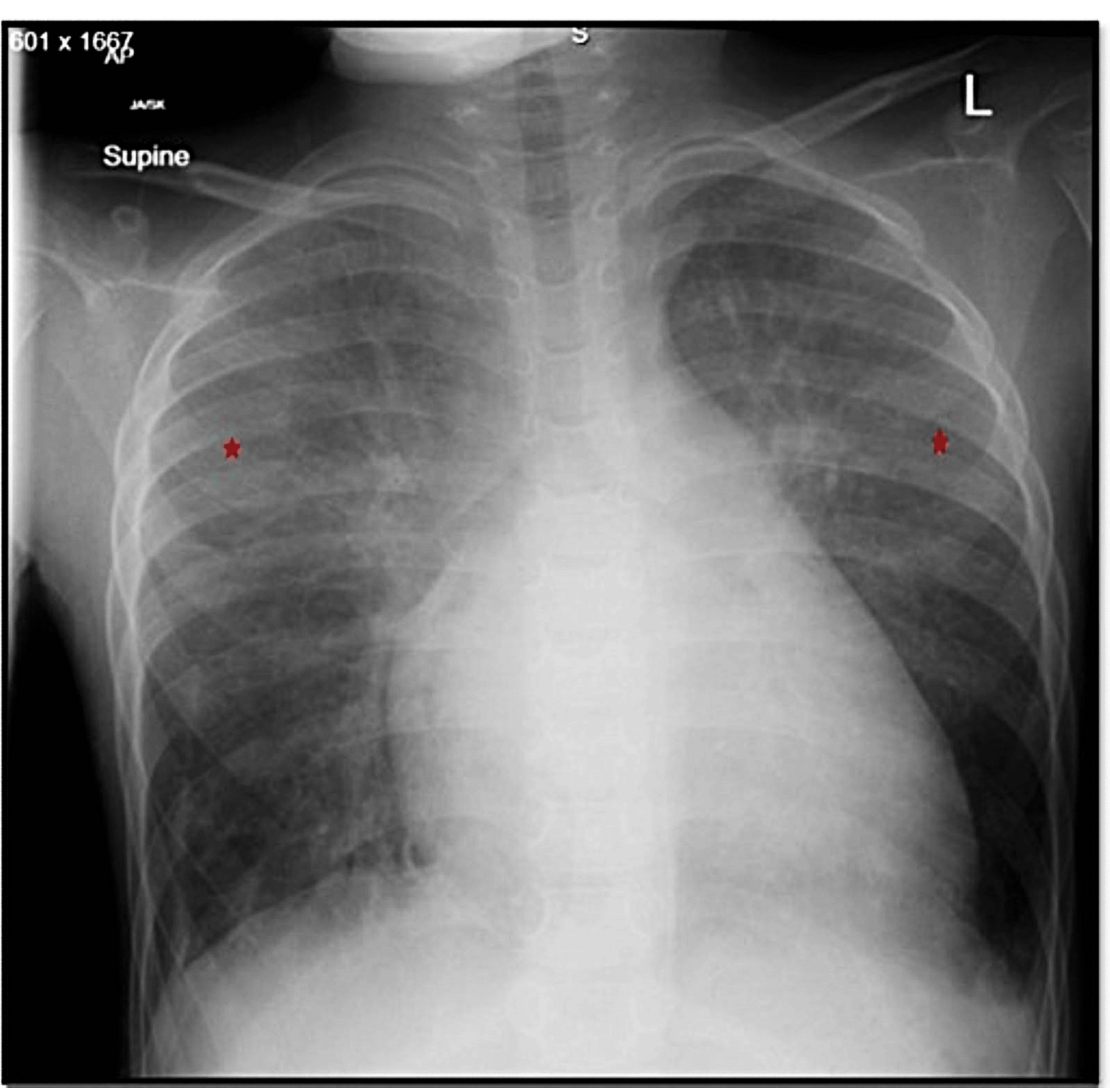
Anteroposterior chest X-ray showing diffuse hazy opacification of the lung parenchyma (red asterix) relatively sparing the peripheral lung bases with medial lung atelectasis.

Laboratory findings were significantly notable of elevated N-terminal pro-B-type natriuretic peptide (NT-pro-BNP) (2177 pg/ml). The remaining labs showed no hematuria, proteinuria, renal dysfunction, or autoantibodies. The reports showed normal bleeding time, prothrombin time, activated partial thromboplastin time, and platelet counts. His chest radiography revealed cardiomegaly and patchy ground-glass opacity in both upper lung zones (Figure 1).

**Figure 2:**
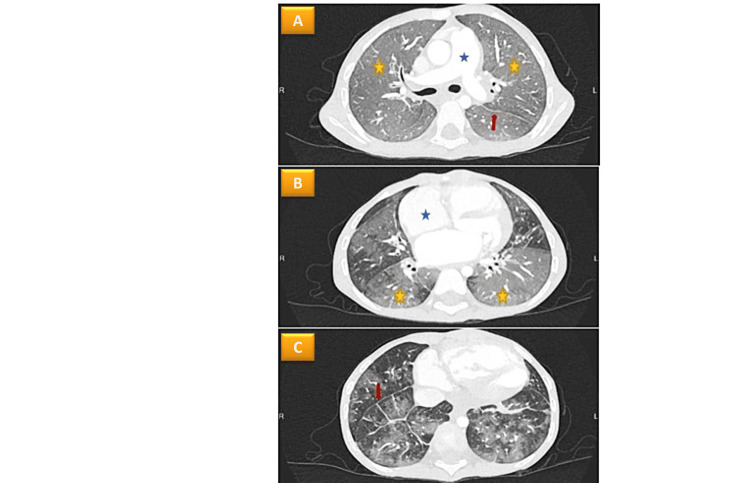
(A) Evidence of widespread pulmonary edema, diffuse alveolar opacification of both lungs (yellow asterix), and basal interlobular septal thickening (red arrow); (B) Dilated right heart without right ventricular hypertrophy; (C) Mild dilatation of the main pulmonary artery.

Cardiac catheterization and echocardiography were performed. There was moderate to severe mitral stenosis and regurgitation and pulmonary arterial hypertension of both pre- and post-capillary phenotype. The mean pulmonary arterial pressure was 35 mmHg, wedged capillary pressure was 15 mmHg, pulmonary vascular resistance was 9.5 wood units, and there was no reversibility with nitric oxide. On the chest CT scan, there was extensive alveolar opacification in both lungs and basal interlobular septal thickening (Figure 2).

**Figure 3:**
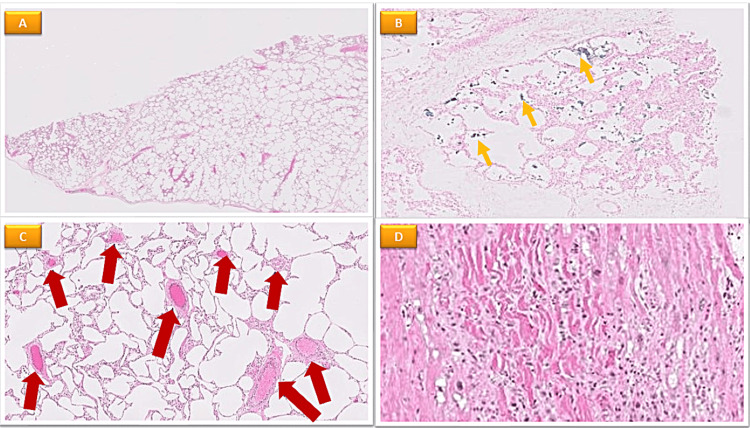
Microscopic description of heart and lung biopsy, showing marked ischemic changes with secondary reparative changes, presumably due to patient's valvular disease (A) The lung tissue shows prominent emphysematous changes; (B) There are foci with a small number of hemosiderin-laden macrophages in the alveolar spaces demonstrated by Perls staining (yellow arrow); (C) Small arteries in the septa show mild-moderate medial hypertrophy and mild interstitial fibrosis (red arrow); (D) Histological sections show disarray of myocardial cells with ischemic/reparative appearances and regenerative nuclei.

A bronchoscopy performed for bronchoalveolar lavage revealed over 97% of the macrophages to be hemosiderin-loaded. Lung tissues were taken out of the top part of the right lower lobe after the mitral valve was repaired. Pathologic testing proved there was secondary pulmonary hemosiderosis (Figure 3).

**Figure 4:**
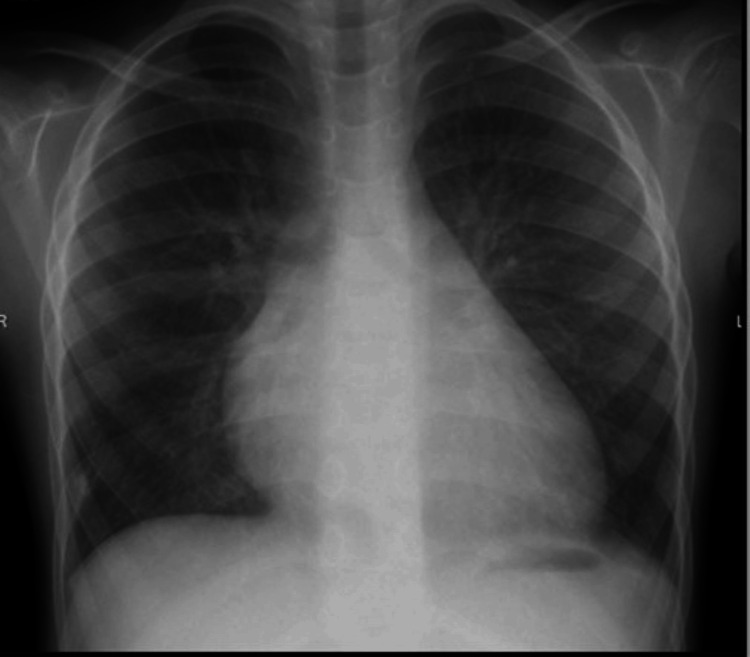
Postoperative anteroposterior chest X-ray showing cardiomegaly and residual mild pulmonary vascular congestive changes.

The patient was given diuretics at first. Heart catheterization followed and his MVS was successfully repaired surgically. Based on the patient's reported clinical improvement, he underwent a follow-up echocardiography and a chest radiograph revealed significant improvement (Figure 4).

## Discussion

Secondary pulmonary hemosiderosis due to congenital MVS is a rare lung condition in children that manifests as a triad of hemoptysis, anemia, and diffuse parenchymal infiltrates on chest radiography [3-5]. Pulmonary hemosiderosis can occur as a primary disease of the lungs or can be secondary to cardiovascular or systemic disease. Primary pulmonary hemosiderosis is more prevalent than secondary forms in children [3].

It is essential to differentiate congenital MVS from rheumatic MVS, as it impacts both treatment and prognosis. Echocardiographic findings are essential in this regard. In congenital MVS, the mitral valve is typically characterized by structural anomalies, such as a parachute mitral valve, double-orifice mitral valve, or supravalvular mitral ring, which differ from the valvular changes seen in rheumatic MVS [9].

The absence of a history of rheumatic heart disease or streptococcal infections, along with negative anti-streptolysin O (ASO) titers, further supports the diagnosis of congenital MVS resulting in secondary pulmonary hemosiderosis. 

The main pathology of pulmonary hemosiderosis in the case of MVS involves chronic venous congestion leading to capillary rupture rather than large-scale arteriole-bronchial anastomosis [7]. As in this instance, pulmonary venous hypertension with secondary pulmonary hemosiderosis can cause gradual declines in exercise tolerance and deconditioning. Exercise limitations have been linked to pulmonary venous hypertension [2]. The patient in the current report did not disclose any cardiorespiratory symptoms or hemoptysis prior to this presentation, other than his inability to engage in routine activities with his friends.

The radiological pattern of secondary pulmonary hemosiderosis due to congenital MVS typically presents as diffuse, patchy ground-glass opacities with inter- or intralobular septal thickening. Mild interstitial thickening can become prominent in long-standing cases, leading to septal fibrosis. Those are in contrast to other causes of ground-glass opacities such as infections (e.g., viral or bacterial pneumonia), interstitial lung diseases (e.g., idiopathic pulmonary fibrosis, hypersensitivity pneumonitis), or acute pulmonary edema [10-11]. The key distinguishing feature in secondary pulmonary hemosiderosis is the presence of hemosiderin-laden macrophages on bronchoalveolar lavage or biopsy, which is absent in most other causes of ground-glass opacities. In our patient, bronchoscopy with bronchoalveolar lavage was carried out prior to the commencement of heart surgery and revealed the presence of hemosiderin-laden macrophages. This condition is also called “brown lung induration” and is characterized histologically by alveolar hemorrhage, hemosiderin-laden macrophages in the alveoli, and, to a lesser extent, in the interstitial. 

Congenital MVS is a broad category of lesions that can develop on their own or in combination with other left heart obstructive conditions. Critical risk factors for survival and long-term outcomes have been shown to include the location of the primary obstructive lesion, the age of presentation, and the presence and severity of pulmonary hypertension [1] The surgical treatment for congenital MVS involves either valve replacement or valvuloplasty [8,9-12]. Following surgical intervention (valve repair or replacement), substantial improvements can be observed in multiple areas, such as exercise capacity, echocardiographic, and resolution of pulmonary hypertension. 

Our patient's condition has much improved, particularly when engaging in activities and exercising with a resolution of pulmonary hypertension by echocardiographic imaging. 

## Conclusions

This case highlights the rare but significant association between congenital MVS and secondary pulmonary hemosiderosis in children. A pediatrician must be vigilant in recognizing subtle signs of progressive exercise intolerance, such as steady activity reduction and broad deconditioning, which may point to underlying cardiac or pulmonary issues. When accompanied by systemic findings like edema or anemia, these signs should prompt an early cardiopulmonary workup. Early diagnosis and timely surgical intervention, as demonstrated in this case, can effectively reverse the disease's progression and improve quality of life. However, it is important to acknowledge that while surgery can improve symptoms and hemodynamics, chronic pulmonary vascular changes, particularly in cases with advanced pulmonary hypertension, may not always be fully reversible. By sharing this unique presentation, we aim to enhance awareness and facilitate the recognition of such complex conditions, ultimately improving outcomes for affected patients. This case also raises the question of whether routine echocardiographic screening in children with unexplained pulmonary hemosiderosis could aid in the earlier detection of MVS and facilitate timely intervention.
